# Perceived Financial Vulnerability Is Related to Perceived Cognitive Impairment and Living Alone

**DOI:** 10.1093/geroni/igab046.336

**Published:** 2021-12-17

**Authors:** Peter Lichtenberg, Maggie Tocco, Juno Moray

**Affiliations:** 1 Wayne State University, Detroit, Michigan, United States; 2 Wayne State University, Wayne State University/Detroit, Michigan, United States

## Abstract

Objective: Perceived financial vulnerability is linked to physical and mental health and also to risk for financial exploitation (Lichtenberg et al., 2020a,b). This study examined the relationship of risk scores for financial exploitation to demographic variables, perceived memory loss and living alone. Methods: The 17-item self-report Financial Exploitation Vulnerability Scale (FEVS) posted on our website https://olderadultnestegg.com was completed by a convenience sample of 258 older adults. Correlational, multiple regression and Chi Square analyses were used. Results: Thirty percent of the sample scored at an elevated risk for financial exploitation due to perceived financial vulnerability. Although this was a convenience sample the results were similar to what was found in a sub-study of the HRS. Thirty eight percent of participants were living alone and 38% reported that their memory was less reliable than a year ago. Financial vulnerability risk score was significantly related to decreased education (r=-.12), living alone (r=.21) and perceived memory loss (r=.35). Eighteen percent of the variance was accounted for in a multiple regression (F(5,250)=10.73, p<0.001, r2=0.18) with all three measures predicting FEVS risk score independently. The combination of perceived memory loss and living alone was significantly associated with the highest percentage of elevated risk scores. Discussion: Perceived financial vulnerability and its relationship to health (e.g. memory loss) and financial exploitation, continues to be under-appreciated in gerontology and geriatrics research. Our findings, consistent with GSA's Longevity Fitness report further highlights this important dimension.

